# Coronary Sinus Lead Removal: A Comparison between Active and Passive Fixation Leads

**DOI:** 10.1371/journal.pone.0153651

**Published:** 2016-04-27

**Authors:** Simon Pecha, Charles Kennergren, Yalin Yildirim, Nils Gosau, Ali Aydin, Stephan Willems, Hendrik Treede, Hermann Reichenspurner, Samer Hakmi

**Affiliations:** 1 Department of Cardiovascular Surgery, University Heart Center Hamburg, Hamburg, Germany; 2 Department of Cardiothoracic Surgery, Sahlgrenska University Hospital, Göteborg, Sweden; 3 Department of Cardiology, Electrophysiology, University Heart Center Hamburg, Hamburg, Germany; 4 Department of Cardiology, St. Adolf-Stift Hospital Reinbek, Reinbek, Germany; Indiana University, UNITED STATES

## Abstract

**Background:**

Implantation of coronary sinus (CS) leads may be a difficult procedure due to different vein anatomies and a possible lead dislodgement. The mode of CS lead fixation has changed and developed in recent years.

**Objectives:**

We compared the removal procedures of active and passive fixation leads.

**Methods:**

Between January 2009 and January 2014, 22 patients at our centre underwent CS lead removal, 6 active and 16 passive fixation leads were attempted using simple traction or lead locking devices with or without laser extraction sheaths. Data on procedural variables and success rates were collected and retrospectively analyzed.

**Results:**

The mean patient age was 67.2 ± 9.8 years, and 90.9% were male. The indication for lead removal was infection in all cases. All active fixation leads were Medtronic^®^ Attain StarFix^™^ Model 4195 (Medtronic Inc., Minneapolis, MN, USA). The mean time from implantation for the active and passive fixation leads was 9.9 ± 11.7 months (range 1.0–30.1) and 48.7 ± 33.6 months (range 5.7–106.4), respectively (p = 0.012). Only 3 of 6 StarFix leads were successfully removed (50%) compared to 16 of 16 (100%) of the passive fixation CS leads (p = 0.013). No death or complications occurred during the 30-day follow-up.

**Conclusion:**

According to our experience, removal of the Starfix active fixation CS leads had a higher procedural failure rate compared to passive.

## Introduction

The number of implanted cardiac resynchronization-therapy devices (CRT) has increased over time, so has the need of CS lead removal [1] [2] [3] [4]. The removal of such leads, indicated by infection or lead dysfunction, can be very complicated, due to the fragile structure of the CS and the possible laceration of the thin vein wall, as supported by animal data [5].

We report our findings on CS lead removal, with the aim to evaluate procedural success related to the mode of CS lead fixation, which has been changing and developing in recent years [6]. Removal of passive fixation leads is usually uncomplicated [7] [8], data regarding removal of chronically implanted active fixation CS leads is however limited. We therefore compared removal of active and passive fixation leads.

## Methods

### Patients

Institutional Review Board approval was obtained from the "Ethikkommision Ärztekammer Hamburg". Written informed consent was given by all patients. We reviewed data from 22 patients who underwent CS lead removal at our center between January 2009 and January 2014. Patients’ characteristics are shown in Table 1. The indication for lead removal was infection in all cases, pocket infection in 18 patients (81.8%) and sepsis in 4 patients (18.2%). All cases were discussed in an interdisciplinary heart team, including a cardiothoracic surgeon and a cardiologist (electrophysiologist).

**Table 1:** Patients’ characteristics.

Patients	AF, n = 6	PF, n = 16	P value
Demographics			
Age, years	71.2 ± 8,9	65.7 ± 10.0	0.25
Male gender, n (%)	6 (100.0)	14 (87.5)	1.00
Body mass index, kg/m^2^	26.4 ± 4.3	28.4 ± 5.2	0.41
Medical history, n (%)			
Prior cardiac surgery	4 (66.7)	11 (68.8)	1.00
Coronary artery disease	3 (50.0)	10 (62.5)	0.65
Diabetes mellitus	3 (50.0)	9 (56.3)	1.00
Hypertension	5 (83.3)	12 (75.0)	1.00
Indication for lead removal, n (%)			
Pocket infection	5 (83.3)	13 (81.3)	1.00
Sepsis	1 (16.7)	3 (18.7)	1.00

AF: active fixation; PF: passive fixation.

### Leads

**Fig 1:**
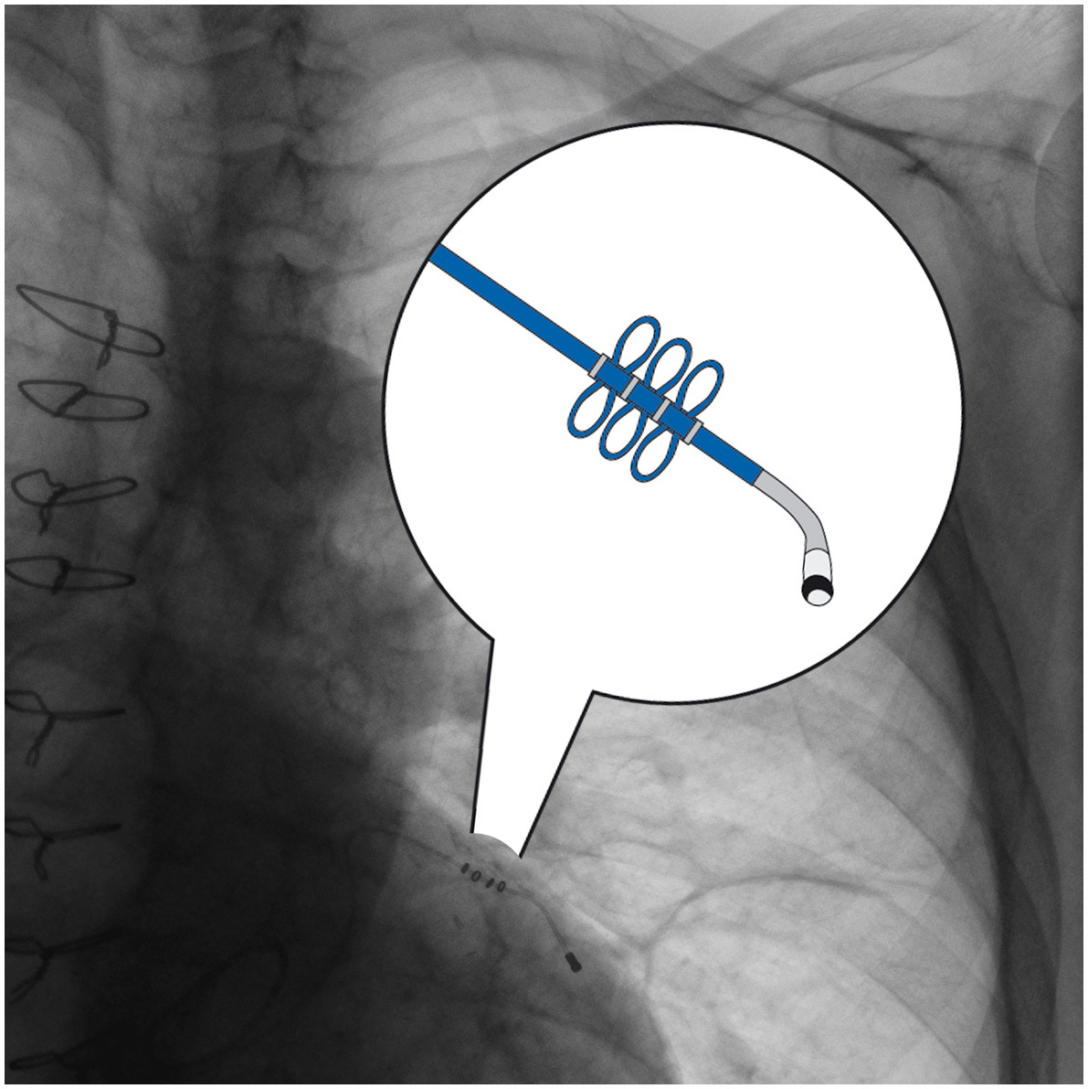
The Attain Starfix active fixation extraction mechanism.

The examined 22 CS leads, were classified in two groups according to the fixation mode. All active fixation leads were Medtronic^®^ Attain StarFix^™^ Model 4195 (Medtronic Inc., Minneapolis, MN, USA). The StarFix CS lead was designed with a new active fixation mechanism. This was achieved using three deployable lobes that enable a stable position in the venous CS branch. The goal of this design was to reduce lead dislocation. Fig 1 shows the StarFix fixation mechanism. The passive fixation leads included ten different models from various manufacturers, which are shown in Table 2.

**Table 2:** Leads and removal outcomes.

Case	Lead model	Fixation	Months	Removal technique	Outcome
1	Medtronic 4195	Active	2.6	Simple traction	Complete
2	Medtronic 4195	Active	1.2	Simple traction	Complete
3	Medtronic 4195	Active	1.0	Simple traction	Complete
4	Medtronic 4195	Active	17.3	LLD, excimer laser	Failure
5	Medtronic 4195	Active	30.1	LLD, excimer laser	Failure
6	Medtronic 4195	Active	7.0	LLD, excimer laser	Failure
7	Boston Scientific 4591	Passive	31.9	Simple traction	Complete
8	Medtronic 4296	Passive	5.7	Simple traction	Complete
9	Medtronic 4193	Passive	106.4	LLD, excimer laser	Complete
10	Medtronic 4196	Passive	24.5	Simple traction	Complete
11	Biotronik 368346	Passive	17.8	Simple traction	Complete
12	Medtronic 4193	Passive	83.5	LLD, excimer laser	Complete
13	Biotronik 354807	Passive	15.0	Simple traction	Complete
14	SJM 1156T	Passive	42.8	Simple traction	Complete
15	SJM 1156T	Passive	38.3	Simple traction	Complete
16	SJM 1055T	Passive	95.2	LLD, excimer laser	Complete
17	Medtronic 4196	Passive	6.1	Simple traction	Complete
18	SJM 1258T	Passive	63.6	Simple traction	Complete
19	SJM 1258T	Passive	32.7	Simple traction	Complete
20	Medtronic 4193	Passive	43.7	Simple traction	Complete
21	Medtronic 4193	Passive	76.9	LLD	Complete
22	SJM 1055T	Passive	91.0	LLD	Complete

SJM: St. Jude Medical; Months: implant duration in months; LLD: lead locking device.

### Removal technique

All procedures were performed in an operating room under general anaesthesia. Continuous arterial blood pressure monitoring was performed via an arterial line placed in the radial artery. A transoesophageal echocardiography probe was placed to monitor for pericardial or pleural effusion. All patients were prepared for emergent sternotomy with cardiopulmonary bypass standby.

The procedures were performed by fluoroscopic guidance. Leads were dissected from the scar tissue and the sleeves were removed. The active fixation mechanism was retracted and simple traction was attempted after insertion of a stylet. Lead locking device was placed in some cases to make the traction procedure more efficient. When the attempt to remove the lead was not successful because of aggressive adhesions, a Spectranetics laser sheath (Spectranetics Corporation, Colorado Springs, CO, USA) was applied up to the level of the CS ostium.

### Definitions

Outcomes were determined according to the recommendations of the Heart Rhythm Society relating to lead removal and defined as follows: 1. Complete procedural success if all targeted leads and all lead material were removed from the vascular space, without any complications or procedure related death. 2. Clinical success if all targeted leads and lead material were removed from the vascular space, but with retention of a small portion of the lead that does not negatively impact the outcome goals of the procedure. 3. Failure if neither complete procedural nor clinical success could be reached [9].

### Statistical analysis

Continuous variables were expressed as mean ± standard deviation and were compared using Student’s *t*-test or the Mann-Whitney test as appropriate Categorical variables were displayed as numbers and percentages and were compared using the chi square test or Fisher’s exact test as appropriate. Statistical analysis was performed using SPSS 21 (SPSS, Inc., Chicago, IL, USA). Statistical significance was defined at p < 0.05.

### Follow-up

Data on procedural variables and success rates, as well as 30-day mortality were obtained by clinical follow-up and collected into a database and analysed retrospectively.

## Results

The cohort was 90.9% male with a mean age of 67.2 ± 9.8 years (range 47–80). Fifteen patients (68.2%) had previous cardiac surgery. Baseline data analysis showed no statistical significant difference between the active and passive fixation patients’ groups.

**Fig 2:**
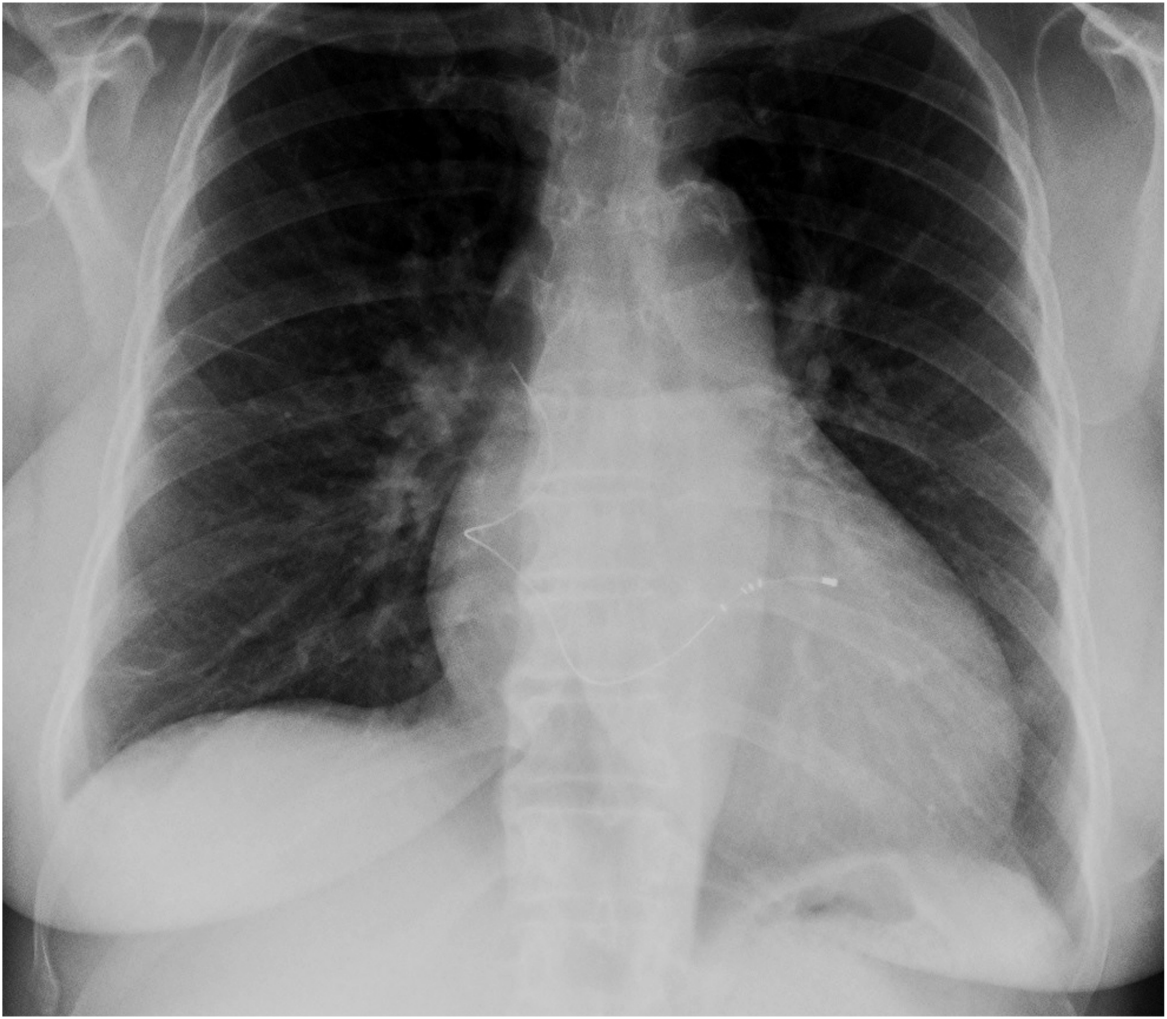
Chest-X-Ray with abandoned LV Lead fragment after extraction failure.

The mean implantation time for the active fixation leads was 9.9 ± 11.7 months (range 1.0–30.1). Retraction of the fixation lobes during the explant procedures was attempted in all six patients, but only in one case was complete retraction successfully achieved (Fig 1 The Attain Starfix active fixation extraction mechanism). A partial retraction of the proximal lobes was noticed in two cases. Only three of six StarFix leads were successfully removed (50%) (Fig 2 Chest-X-Ray with abandoned LV Lead fragment after extraction failure). Those were leads that were implanted for a mean time of 1.6 months (1.0–2.6 months). The leads where the extraction attempt failed were implanted for a mean duration of 18.1 months 7–30.1 months) Laser sheath application was required in three cases (50%). (Table 2)

The mean time from initial implantation for the passive fixation leads was 48.7 ± 33.6 months (range 5.7–106.4), resulting in significantly longer implant duration compared to the active fixation group (p = 0.012). All passive fixation leads were successfully removed (100%), showing a significantly higher procedural success rate compared to the active fixation group (100% versus 50%, p = 0.013). Lead locking device assistance was needed in two cases (12.5%) and an excimer laser was applied in three cases (18.75%). (Table 2)

There were no procedural complications as a result of CS lead removal, and no death occurred during 30-day follow-up.

Re-implantation status is given in Table 3. A CS lead re-implantation was successfully implemented transvenously in 3 (50%) patients of the active fixation group and in 12 (75%) patients of the passive fixation group. Left ventricular epicardial lead implantation was performed in 3 (50%) patients of the active fixation group and in 3 (18.75%) patients of the passive fixation group when the transvenous approach was unsuccessful. Biventricular pacing was no longer needed in one patient (6.25%) of the passive fixation group because of non-response to the resynchronization-therapy. The three patients with active lead extraction failure received extended antibiotic treatment. After antibiotic treatment, the infection parameters were normalized. In all three patients, an epicardial CS lead implantation was performed. No patient with remaining lead fragment had a recurrent infection during mean follow-up period of 13 +/-3 months.

**Table 3:** Re-implantation status.

Leads	n = 22
Active fixation, n (%)	
Transvenous CS lead re-implantation	3 (13.6)
Left ventricular epicardial lead implantation	3 (13.6)
Passive fixation, n (%)	
Transvenous CS lead re-implantation	12 (54.6)
Left ventricular epicardial lead implantation	3 (13.6)
Re-implantation was no longer needed because of non-response to CRT	1 (4.6)

CS: coronary sinus; CRT: cardiac resynchronization-therapy.

## Discussion

We present our CS lead removal experience in patients with CRT devices. All of these patients also had right atrial and right ventricular leads present at the time of removal. Our experience with removal of passive fixation CS leads indicates that removal is straightforward unless strong calcifications or aggressive adhesions with other existing leads are present. We have successfully removed 16 passive fixation CS leads. Eleven of these passive fixation CS leads were explanted after insertion of a stylet, using only simple traction. Lead locking devices with or without laser sheaths were required to remove the remaining five leads.

We also were able to successfully explant three active fixation CS leads with short implant duration. Those patients had a previous implanted dual-chamber implantable cardioverter defibrillator that was later upgraded to a cardiac resynchronization-therapy defibrillator and underwent an early lead removal for systemic or local device infection. Neither complete procedural or clinical success could be achieved in the remaining three cases of the active fixation CS leads with mean implant duration of 18.1 months.

Limited experiences with active fixation CS lead removal have been reported, especially with chronically implanted leads [10]. Removal of old active fixation CS leads can be extremely challenging as previously described in case reports [11] [12] [13] [14].

The active fixation CS lead (Medtronic^®^ Attain StarFix^™^ Model 4195) is difficult to remove from the CS, due to fibrotic tissue ingrowth between the fixation lobes. As previously described by Baranowski et al. this growth can lead to extraction failures [11]. The removal of StarFix leads is basically identical to other leads; however, in our opinion it is preferred not to use mechanical dilator or laser sheath deep in the CS. The risk of tearing these cardiac veins is very high and may lead to an acute bleeding into the pericardial space with subsequent tamponade.

Kypta et al. described a case of CRT related infection requiring removal of a StarFix lead. Although the active fixation lobes could not be released, the StarFix lead was successfully removed from the CS with a mechanical dilator sheath, which was advanced into the lateral coronary vein. However, immediately post-procedure a discrete pericardial effusion, without need for intervention, was seen [12].

Our results extend the findings of Williams et al., they removed successfully 59 passive fixation leads. Only one extraction failure with a 26.5 months old active fixation StarFix lead was reported. No complete lobe retraction was achieved. Despite the use of laser sheath into the CS, the complete procedural success could not be accomplished [13].

Naegele et al. faced similar problematic with removal of one StarFix lead implanted for less than two months. The retraction of the deployable lobes and the removal of the lead were not possible [14].

However, it has to be admitted that Cronin et al. and Maytin M et al. have reported good procedural outcomes in active fixation lead removal, with advancement of the laser sheath into the CS [15] [16].

In our series, three failure cases of active fixation StarFix leads were observed. In cases 4 and 5 (Table 2) with implant duration of 17.3 and 30.1 months, a retraction of the deployable lobes was not achieved. Despite the use of laser sheath assistance, extraction of the leads was not possible and they were capped and abandoned. In a further case No. 6 (Table 2) with implant duration of 7 months, only partial retraction of the proximal lobes was achieved. During counter traction the lead fractured, leaving a tip of more than 4 cm in situ. However, it has to be admitted that we did not use laser sheaths or mechanical extraction tools deep in the CS, being afraid of a markedly increased procedural risk.

The issue of being more aggressive (eg. using powered sheath inside the CS and branches) or more conservative (eg. abandoning the lead) have to be further investigated. The operator here has to balance the risk of leaving the lead expecially in case of infection and the risk of being more aggressive with the possibility of important clinical consequences like perforation and cardiac tamponade. However, to date there is not much evidence concerning this issue and in future, more studies including larger patient numbers are needed.

First reports with the extraction of the new Medtronic 20066 Attain Stability lead have been published. This LV lead has a retrievable helix fixation which might enable for easier extraction. Bontempi et al have report a case of an Attain Stability active fixation lead which has been extracted after implant duration of 8 months without difficulties after [17]. However this is results have to be confirmed in larger patient populations and in leads with longer implant duration.

Our results show that removal of passive fixation CS leads is possible with excellent results. In most cases, simple traction alone enables removal of these leads, but in some cases specific extraction tools are required. However, in contrast, removal of active fixation leads remains difficult and is associated with a limited procedural success rate. Unless the operator and patient are willing to accept the markedly increased risk of passing the CS ostium with mechanical devices or laser sheaths, extraction might especially for chronically implanted active fixation leads not be possible. This may result from adhesions and fibrotic tissue growth between the fixation lobes of these leads.

### Limitations

This study is a retrospective analysis and our series is limited by its small size. Furthermore, it is a single-center study and the removal procedures were performed by two experienced operators, which may influence the results of the study compared to other centers. The different implantation times of both lead groups may have influenced the removal results. Moreover, the lack of long-term follow-up and the possible late complications could be undetected.

## Conclusions

The removal of StarFix active fixation leads can, even after short implant duration, be complicated or sometimes impossible. In our opinion, active fixation leads should be used with caution and we suggest that other options have to be evaluated before the use of these leads, especially in patients with a long life expectancy or in patients with high risk of device infection.

## Supporting Information

S1 Table Patient and lead demographics. (XLSX)
